# Effectiveness of serum beta-2 microglobulin as a tool for evaluating donor kidney status for transplantation

**DOI:** 10.1038/s41598-020-65134-6

**Published:** 2020-05-15

**Authors:** Sung Whan Cha, In Sik Shin, Deok Gie Kim, Sung Hoon Kim, Jun Young Lee, Jae Seok Kim, Jae Won Yang, Byoung-Geun Han, Seung Ok Choi

**Affiliations:** 1 Department of Surgery, Yonsei University Wonju College of Medicine, Wonju Severance Christian Hospital, Wonju, South Korea; 2 Internal Medicine, Yonsei University Wonju College of Medicine, Wonju Severance Christian Hospital, Wonju, South Korea

**Keywords:** Risk factors, Kidney

## Abstract

Kidney transplantations using expanded criteria donors (ECD) are being increasingly adopted, but no consensus tools are available to evaluate donor kidney status. Beta-2 microglobulin (B2MG) is a marker of kidney function, and herein, we evaluate the usefulness of assessing B2MG to evaluate donor kidney status. Fifty-seven kidney transplantations were performed from March 2017 to April 2019. Medical records were retrospectively reviewed, and relationships between clinical and laboratory variables and transplant outcomes were investigated. Thirty-eight patients received a standard criteria donor kidney and 19 patients an ECD kidney. Ten patients experienced delayed graft function (DGF), but no patient experienced primary nonfunction. Of the parameters studied, only donor renal replacement therapy (RRT) [odds ratio (OR) 24.162; *p* = 0.018] and donor serum B2MG (OR 22.685; *p* = 0.022) significantly predicted DGF. The presence of either of these two risk factors can better reflect the condition of the donor than previous classification. However, on their last follow-up creatinine and estimated glomerular filtration rate values in those with or without these risk factors were not significantly different. For an ECD with a B2MG level of <7.18 and no history of RRT, kidney transplantation can be undertaken without considering the possibility of kidney discard.

## Introduction

Chronic kidney disease (CKD) is becoming a major global health issue because of its prevalence and economic cost. Societal aging and the associated increases in the prevalence of hypertension and diabetes inevitably mean that the number of CKD patients will continue to increase^1^. Renal replacement therapies such as dialysis or kidney transplantation (KT) are considered proper management options for patients with severe CKD or end-stage renal disease (ESRD), and KT is considered a better choice than dialysis in many respects^2,3^. Furthermore, as demand for KT increases, so does interest in the prognosis of patients with transplanted kidneys in terms of quality of life and cost-effectiveness.

It is practically impossible to provide KT to all indicated patients due to donor shortages. To overcome this situation, kidneys from expanded criteria donors (ECDs) or donors after cardiac death (DCDs) have been used worldwide^4,5^, but this has also inevitably increased discard rates^6^. Even patients fortunate enough to have undergone KT may experience primary nonfunction, delayed graft function (DGF), or rejection, and frequently, these conditions eventually result in poor graft outcomes or early graft failure. Furthermore, these conditions have been associated with prolonged hospitalization, higher costs, and mortality^7,8^.

Many clinical and laboratory risk factors have been evaluated in attempts to predict the status of donor kidneys, but discard and DGF rates have not substantially changed. Accordingly, we considered the possibility of using novel predictors of kidney status and focused on assessing donor beta2-microglobulin (B2MG) serum level. It has been reported that the ability of serum B2MG to predict renal failure in the general population and CKD patients is unaffected by sex, age, or race. Also, the estimated glomerular filtration rate (eGFR) and calculated serum B2MG level have been reported to reflect renal function well. However, donor serum B2MG has not been evaluated in the context of the association between transplant outcomes and ECD and DCD kidneys.

## Results

### Donor characteristics

Of the 57 recipients, 38 (66.7%) received a standard criteria donor (SCD) kidney and 19 (33.3%) an ECD kidney. Gender ratios and body mass indices (BMIs) were similar in the SCD and ECD groups. The frequency of acute kidney injury (AKI) > grade 2 was 11 (28.9%) in the SCD group and 4 (21.1%) in the ECD group (*p* = 0.523). The numbers of donors that received renal replacement therapy (RRT) in the SCD and ECD groups were 6 (15.8%) and 1 (5.3%), respectively (*p* = 0.405). Initial creatinine (Cr), final Cr, highest Cr values, urine and serum cystatin C, and urine and serum B2MG levels were not significantly different between the two groups. The baseline characteristics of donors in the two groups are displayed in Table 1.

**Table 1:** Demographic and laboratory characteristics of donors.

Variables		SCD (n = 38)	ECD (n = 19)	*p*
Age, year		40 (1–59)	60 (50–74)	<0.001
Gender	Female	14 (36.8%)	9 (47.4%)	0.445
	Male	24 (63.2%)	10 (52.6%)	
BMI, kg/m^2^		24.2 (18.4–33.6)	24.5 (19–30.1)	0.644
Hypertension, n (%)		2 (5.3%)	12 (63.2%)	<0.001
Diabetes, n (%)		0 (0%)	4 (21.1%)	0.01
HbA1C, %		5.5 (4.6–6.6)	5.6 (5.2–7.4)	0.08
Initial Cr, mg/dL		0.97 (0.4–6.47)	1.1 (0.46–1.72)	0.771
Initial eGFR, mL/min/1.73 m^2^		81.1 (10.3–221.0)	70.1 (43.8–151.1)	0.156
Final Cr, mg/dL		0.97 (0.36–5.06)	0.91 (0.54–3.59)	0.497
Final eGFR, mL/min/1.73 m^2^		80.3 (13––233.3)	77.3 (18.9–167.6)	0.633
Highest Cr, mg/dL		1.19 (0.51–6.47)	1.34 (0.64–3.65)	0.42
Urine cystatin C		0.11 (0.03–6.67)	1.1 (0.03–16.2)	0.089
Serum cystatin C, mg/L		0.92 (0.56–3.43)	1.11 (0.68–2.68)	0.833
Cystatin C GFR, mL/min		88.8 (26.7–143.3)	74.3 (33.1–118.7)	0.372
Urine B2MG		0.23 (0.02–96.13)	14.14 (0.03–82.64)	0.123
Serum B2MG, mg/L		2 (0.09–14.22)	4.03 (1.08–44)	0.177
AKI more than grade 2		11 (28.9%)	4 (21.1%)	0.523
RRT, n (%)		6 (15.8%)	1 (5.3%)	0.405
Cause of death, n (%)	Living	17 (44.7%)	3 (15.8%)	0.106
	Hypoxia	7 (18.4%)	7 (36.8%)	
	Cerebral	13 (34.3%)	7 (36.8%)	
	Cardiac	1 (2.6%)	2 (10.6%)	

SCD, standard criteria donor; ECD, expanded criteria donor; BMI, body mass index; Cr, Creatinine; eGFR, estimated glomerular filtration rate; B2MG, beta-2 microglobulin; AKI, acute kidney injury; RRT, renal replacement therapy eGFR was calculated using the Modification of Diet in Renal Disease four-variable equation.

### Recipient characteristics and outcomes

The demographic characteristics of the recipients, including age, gender, BMI, primary kidney disease, RRT type and duration before KT, were similar in the SCD and ECD groups, and the HLA mismatch and immunologic risk numbers, kidney weights, and cold ischemic times (CIT) were also similar. Ten recipients experienced DGF; 8 (21.1%) in the SCD group and 2 (10.5%) in the ECD group (*p* = 0.469). Rates of biopsy-proven rejections were also similar. However, last follow-up median Cr (1.09 mg/dL) and eGFR (68.5 mL/min) were better in the SCD group than in the ECD group (1.41 mg/dL and 54 mL/min, respectively) (*p* = 0.055, 0.003, respectively). Baseline characteristics of recipients of SCD and ECD kidneys are shown in Table 2.

**Table 2:** Demographic and laboratory characteristics of recipients and transplantation outcomes.

Variables		SCD (n = 38)	ECD (n = 19)	*p*
Age, year		53 (19–65)	54 (36–65)	0.075
Gender	Female	12 (31.6%)	9 (47.4%)	0.244
	Male	6826 (71.4%)	10 (52.6%)	
BMI, kg/m^2^		23.7 (13.9–32.6)	24 (18.5–32.4)	0.809
Primary kidney disease	DM	8 (21.1%)	5 (26.2%)	0.513
	HTN	5 (13.2%)	4 (21.1%)	
	GN	14 (36.8%)	3 (15.8%)	
	Other*	4 (10.5%)	3 (15.8%)	
	Unknown	7 (18.4%)	4 (21.1%)	
Prior treatment, n (%)	Preemptive	13 (34.2%)	1 (5.3%)	0.124
	HD	16 (42.1%)	15 (78.9%)	
	PD	3 (7.9%)	2 (10.5%)	
	2nd graft	6 (15.8%)	1 (5.3%)	
Time on dialysis, month		32 (0–210)	36 (0–216)	0.644
HLA mismatch		4 (0–6)	4 (2–5)	0.134
Immunologic risk	Low	15 (39.5%)	11 (57.9%)	0.188
	High	23 (60.5%)	8 (42.1%)	
Kidney weight, gram		217 (124–315)	251 (165–300)	0.086
CIT, min		145 (35–754)	158 (58–228)	0.93
DGF		8 (21.1%)	2 (10.5%)	0.469
Acute rejection, n (%)	ACR	1 (2.6%)	2 (10.5%)	>0.999
	ABMR	4 (10.5%)	5.31 (11.1%)	
Last follow-up Cr, mg/dL		1.09 (0.52–3.64)	1.41 (0.82–5.98)	0.055
Last follow-up eGFR, mL/min/1.73 m^2^		68.5 (14.5–156.3)	54 (11.5–78.3)	0.003
Graft survival, n (%)		37 (97.4%)	18 (94.7%)	>0.999
Patients survival, n (%)		34 (89.5%)	17 (89.5%)	>0.999

SCD, standard criteria donor; ECD, extended criteria donor; DM, diabetes mellitus; GN, glomerulonephritis; BMI, body mass index; HD, hemodialysis; PD, peritoneal dialysis; HLA, human leukocyte antigen; CIT, cold ischemic time; DGF, delayed graft function; ACR, acute cellular rejection; ABMR, antibody-mediated rejection; Cr, creatinine; eGFR, estimated glomerular filtration rate.

*Other includes renal tuberculosis, cystic kidney disease, lupus nephritis, nephrotoxic agents.

### Risk factors of delayed graft function

Receiver operating characteristic (ROC) curve analysis showed donor final eGFR, highest Cr, serum cystatin C, serum B2MG level, and cold ischemic time significantly predicted DGF. Areas under curves (AUCs) of parameters are displayed in Table 3. Univariate logistic regression analysis revealed hypoxic-origin brain death, donor RRT, an AKI grade > 2, and donor serum cystatin C and serum B2MG levels significantly predicted DGF. However, multivariate logistic regression analysis revealed that donor RRT [odds ratio (OR) = 12.837; *p* = 0.025; 95% confidence interval (CI) = 1.374 - 119.954] and donor B2MG level of ≥ 7.18 mg/L (OR = 12.719; *p* = 0.011; 95% CI = 1.785–90.63) were significant independent risk factors of DGF. Logistic regression analysis results are provided in Table 4.

**Table 3:** Receiver operating characteristic curve analysis of continuous variables for the prediction of DGF.

Variables	Cuff-off value	AUC	*p*	Sensitivity	Specificity
Initial creatinine, mg/dL	>1.04	0.635	0.261	71.4%	56.8%
Initial eGFR, mL/min/1.73 m^2^	<79.6	0.545	0.66	51.1%	70.0%
Final creatinine, mg/dL	>1.77	0.853	0.003	85.7%	78.4%
Final eGFR, ml/min/1.73 m^2^	<69.1	0.673	0.087	72.3%	70.0%
Highest creatinine, mg/dL	>2.8	0.857	0.003	85.7%	89.2%
Serum cystatin C, mg/L	> 1.7	0.851	0.004	85.7%	86.5%
Serum beta-2 microglobulin, mg/L	>7.18	0.896	0.001	85.7%	86.5%
Kidney weight, gram	>251	0.755	0.034	71.4%	70.3%
Cold ischemic time, min	>184	0.803	0.012	71.4%	73.0%

DGF, delayed graft function; AUC, area under the curve; eGFR, estimated glomerular filtration rate.

**Table 4:** Analysis of independent risk factors of delayed graft function.

Variables		Univariate logistic analysis			Multivariate logistic analysis		
		OR	*p*	95% CI	OR	*p*	95% CI
Donor type	ECD	0.441	0.334	0.084–2.32			
	Deceased	6.667	0.087	0.78–56.968			
Origin of brain death	Hypoxic *vs*. living	38	0.023	1.659–870.452			
RRT of donor	Yes	22.5	0.001	3.426–147.779	12.837	0.025	1.374–119.954
AKI	More than grade 2	6.333	0.013	1.472–27.242			
Final creatinine, mg/dL	≥ 1.77	3.923	0.059	0.951–16.188			
Highest creatinine, mg/dL	≥ 2.8	2.417	0.215	0.599–9.753			
Serum cystatin C, mg/L	≥ 1.7	17	0.002	2.897–99.759			
Serum beta-2 microglobulin, mg/L	≥ 7.18	21	0.001	3.494–126.213	12.719	0.011	1.785–90.63
Kidney weight, gram	≥ 251						
Cold ischemic time, min	≥ 159.5	2.417	0.215	0.599-9.753			
Immunologic risk	High	2.236	0.283	0.515–9.71			

OR, odds ratio; CI, confidential interval; ECD, expanded criteria donor; RRT, renal replacement therapy; AKI, acute kidney injury; eGFR, estimated glomerular filtration rate.

### Comparison of the baseline characteristics of recipients reclassified based on the risk factors

Contrary to the traditional SCD/ECD classification, DGF developed more frequently in those recipients with a risk factor (2, 5.4% *vs*.7, 50%; *p* = 0.001). All living donor kidney recipients belonged to the group without a risk factor. Laboratory parameters including initial eGFR, highest Cr, final Cr, and final eGFR were much better in the group without risk factors than in the group with risk factors, and the prevalence of DGF and biopsy-proven rejection were lower in recipients without a risk factor. However, recipient last follow-up Cr and eGFR values were similar in the two groups. A summary of the results is provided in Table 5.

**Table 5:** Demographic and laboratory characteristics according to the presence of risk factors*.

Variables		No (n = 37)	Yes (n = 14)	*p*
DGF	No	35 (94.6%)	7 (50%)	0.001
	Yes	2 (5.4%)	7 (50%)	
SGF	No	22 (59.5%)	2 (14.3%)	0.004
	Yes	15 (40.5%)	12 (85.7%)	
Induction agent	Basiliximab	25 (67.6%)	4 (28.6%)	0.012
	ATG	12 (32.4%)	10 (71.4%)	
Immunologic risk	Low	22 (59.5%)	3 (21.4%)	0.203
	High	15 (40.5%)	11 (78.6%)	
Donor criteria	SCD	25 (67.6%)	7 (50%)	0.247
	ECD	12 (32.4%)	7 (50%)	
	Living	19 (51.4%)	0 (0%)	0.001
	Cadaver	18 (48.6%)	14 (100%)	
Rejection	No	935 (84.6%)	10 (71.4%)	0.015
	Yes	2 (5.4%)	4 (28.5%)	
Hypertension, n (%)		10 (27%)	3 (21.4%)	>0.999
Diabetes, n (%)		2 (5.4%)	1 (14.3%)	0.3
Initial creatinine, mg/dL		0.87 (0.51–2.03)	1.27 (0.46–6.47)	0.159
Initial eGFR, mL/min		83.9 (41.8–157.6)	64.5 (10.3–151.1)	0.029
Highest creatinine, mg/dL		1.09 (0.51–3.18)	3.22 (1.28–6.47)	<0.001
Final creatinine, mg/dL		0.81 (0.36–2.76)	2.57 (0.79–4.41)	0.001
Final eGFR, mL/min/1.73 m^2^		89.8 (25.8–233.3)	24.6 (13.4–78.6)	<0.001
Urine B2MG		0.23 (0.02 – 96.13)	14.97 (0.08 – 82.64)	0.107
AKI more than grade 2		4 (10.8%)	6 (85.8%)	<0.001
HLA mismatch		4 (2–5)	5 (2–5)	0.351
Cold ischemic time, min		135 (35–228)	181 (143–263)	<0.001
Last follow-up creatinine, mg/dL		1.15 (0.52–5.98)	1.25 (0.6–5.36)	0.249
Last follow-up eGFR, mL/min		63.8 (7.7–127.1)	57.2 (11.5–156.3)	0.471
Graft failure, n (%)		0 (0%)	1 (7.1%)	0.275

DGF, delayed graft function; SGF, slow graft function; ATG, anti-thymocyte globulin; SCD, standard criteria donor; ECD, expanded criteria donor; eGFR, estimated glomerular filtration rate; B2MG, beta-2 microglobulin; AKI, acute kidney injury; HLA, human leukocyte antigen.

* Risk factors were receipt of renal replacement therapy and a serum beta-2 microglobulin level ≥ 7.18 mg/L.

## Discussion

In this retrospective study, we analyzed the incidence and risk factors of DGF during the immediate postoperative period after KT. DGF is the most common problem after deceased donor KT but is also encountered after living donor KT. The main contributors to the development of DGF are ischemia-reperfusion injury and hemodynamic instability^9^. The likelihood of DGF is the most important consideration when a surgeon decides to use or discard a kidney with AKI from an ECD. Several authors have reported that KT using an ECD with AKI results in comparable short- and long-term outcomes^10–14^, although other studies have shown contrary results^15^. Although many authors have reported it is acceptable to use an ECD kidney with AKI^16^, no practical tools are available to evaluate ECD kidneys with AKI.

In this study, we reviewed several risk factors believed to result in DGF. Of these, only donor RRT and B2MG were observed to be related to the development of DGF. Donor RRT means that the donor has a kidney with an AKI > grade 3 according to the Kidney Disease Improving Global Outcomes (KDIGO) definition. Even though many studies have reported that KT using an ECD with AKI have outcomes comparable to those using an ECD without AKI or SCD, subgroups that needed RRT or showed Cr level elevation showed poor outcomes^10,17,18^. A significant risk factor of primary nonfunction or poor graft outcome in KT was severe AKI that needed RRT^16^. However, our results showed that RRT was the only risk factor of DGF and did not affect short-term outcomes. Unlike our study, previous studies have included data from multiple centers and donors from multiple centers, and those previous studies did not precisely reveal the final Cr level of donors and estimate AKI status. In our study, although we examined a small number of cases, all donors were procured in our hospital, and all laboratory results were reported by using a sample collected on procurement day. These factors may explain why there are differences in the primary nonfunction incidences and effects on graft outcome between our study and previous studies. B2MG has a central role in cellular immunology, is related to residual kidney function, and its plasma concentration is correlated with GFR.^19^ However, careful interpretation of a serum B2MG increase is needed as a B2MG increase can occur in solid organ malignancy, lymphoproliferative and autoimmune diseases^20–23^, and inflammatory bowel disease^24^. B2MG is associated with the major histocompatibility complex I (MHC-I) in all nucleated cells, and under the above conditions, a large number of cells may be generated and shed B2MG^25^. Furthermore, brain death induces a profound inflammation reaction, which influences multiple transplantable organs and causes kidney tubular damage by generating cascades of activated cytokines^26,27^. Ischemic-reperfusion injury can induce similar events. B2MG is an indicator of systemic inflammation, but not of kidney status, and the outcomes and functions of transplantable organs are significantly influenced by inflammation reactions. Thus, B2MG status, which is strongly correlated with GFR and inflammation, may be a meaningful marker of transplantable organ function, including kidney function. Most B2MG is reabsorbed in proximal tubules, and thus, a high level of urinary B2MG indicates tubule damage^25^. However, Cooper *et al*.^28^ reported that acute tubular injury did not affect transplant outcome. In the present study, urinary B2MG was not predictive of DGF, and the urinary B2MG level was not different between the SCD and ECD groups. In a previous study, the effectiveness of serum B2MG level as a biomarker of adverse outcomes in CKD was investigated. In patients with ESRD or chronic allograft damage after renal transplantation, a previous study reported that compared to urinary B2MG level, serum B2MG level predicted kidney status more accurately^25^. However, there are no reports evaluating donor kidney status or predicting the incidence of DGF by assessing serum B2MG levels. Even though our study was conducted at a single institution and on a small number of patients, serum B2MG showed good predictive power for DGF with RRT, and it reflected the condition of donors more accurately than those derived by using the SCD and ECD classification approaches.

DGF is not an uncommon complication during the period immediately following transplantation and is associated with prolonged hospitalization, increased cost, and the possibility of graft failure^29^. If identification of DGF risk factors before transplantation were possible, recipient outcomes would be improved, and donors would have an additional incentive to prevent kidney discard. The therapeutic interventions used to prevent DGF have been widely investigated and reviewed. A recent Cochrane review showed that machine perfusion provides a better means of preserving donor kidneys than cold storage in terms of preventing DGF and improving outcome^30^. Modifications of immunosuppressant agents have been investigated, and the selection of induction agents is a well-debated issue. Rabbit anti-thymocyte globulin (rATG) produced better results than interleukin-2 receptor blockers in some studies^31,32^, but other studies produced contradictory results^33^. In the present study, rATG was used to a greater extent in the recipients with risk factors group, and its use was not associated with reductions in DGF and rejection rates. However, final creatinine and eGFR levels in the recipients with risk factors group were similar to those in the recipients without risk factor group despite the presence of DGF and rejection. Even though machine perfusion can produce better results, many institutions cannot use machine perfusion due to resource limitations. Thus, modulation of immunosuppressant agents may be a more realistic option in many situations.

In conclusion, we increased the use of ECDs and DCDs to address donor shortage, which means we now attach greater importance to detecting patients at risk to prevent DGF and improve long-term outcomes. In this regard, it is important to find kidney function indicators that can be quickly and repeatedly assessed. Although our study was conducted on a small cohort at a single institution, the results show that if serum B2MG is <7.18 mg/L and therapeutic, and there is no history of donor RRT, KT from an ECD with AKI can be performed without the need to consider kidney discard.

## Methods

### Patients

From March 2012 to July 2019, 135 patients underwent KT at Wonju Severance Christian Hospital, Wonju, South Korea, with all transplants performed by a single surgeon. B2MG samples were prospectively obtained beginning in March 2017, and the present retrospective study was performed using the data of 57 patients that registered from March 2017 to April 2019. This study was approved beforehand by the Institutional Review Board of Yonsei University Wonju Severance Hospital (CR318083). The requirement for informed consent was waived because of the retrospective nature of the study. All studies were conducted according to the principles of the Declaration of Helsinki. All living and deceased donors were evaluated for infections of hepatitis B or C virus (HBV, HCV) or human immunodeficiency virus (HIV) before transplantation. Transplantation was conducted in cases when donors did not show evidence of HBV, HCV, and HIV infections. All procurements and transplantations were performed by surgeons of our departments who were registered into Korean Network for Organ Sharing (KONOS), under the permission of KONOS. There is no procurement from prisoners.

### Variables

Samples of blood and urine to assess serum cystatin C, creatinine, B2MG, and urine cystatin C levels were collected and evaluated one day before procurement of a deceased donor or living donor kidney. B2MG level was measured by enzyme-linked fluorescent assay (ELFA) using a Vidas analyzer (bioMérieux, France). The eGFR was calculated using the Modification of Diet in Renal Disease four4-variable equation^34^. SCD, ECD, DGF, and acute AKI were defined as we described in a previous article^13^.

Recipients at immunologic risk were defined as those that had undergone a second or ABO-incompatible KT, had a panel reactive antibody level > 20%, or had received a kidney from a deceased donor with an AKI grade > 3.

### Immunosuppressive agents

When neither donor nor recipient had any risk factor, we chose basiliximab as the induction agent. Briefly, 20 mg of basiliximab were injected on the day of the operation and again at four days after surgery. However, when a recipient was deemed high immunologic risk, rATG was the induction agent and was administered at 4.5 mg/kg/day in the first three days. We used a triple regimen of tacrolimus, corticosteroid, and mycophenolate mofetil as the maintenance immunosuppressant agents. Trough levels of tacrolimus and mycophenolate mofetil dosage were controlled as we previously described^13^ with the exception that corticosteroid administration was started as 500 mg of methylprednisolone followed by dose tapering as previously described^13^. We checked cytomegalovirus (CMV) and BK virus infection status repeatedly. CMV infection status was checked postoperatively at 1 day, 1 week, 1 month, and 3 months. The BK virus status was checked postoperatively every three months for 1 year and then every 6 months. If quantitative CMV and BK virus test results were positive, we performed qualitative tests. As needed, oral valganciclovir was used to treat CMV infection, and a reduced level of immunosuppressive agents was applied for BK virus treatment.

### Outcomes

The baseline characteristics of SCDs and ECDs were compared and their effects on transplantation outcomes were investigated. Because there was no case of primary nonfunction, we performed analysis to identify risk factors for DGF. We also compared recipient baseline characteristics were compared and transplantation outcomes analyzed according to the absence or presence of risk factors.

### Statistics

Statistical analyses were performed using SPSS Ver. 25.0 (SPSS Inc., Chicago, IL, USA). Continuous variables are presented as medians and ranges, and categorical variables as numbers and percentages. Categorical and continuous variables were compared using Fisher’s exact t test and the *t*-test, respectively. Cut off values of factors predictive of DGF were determined by performing receiver operating characteristic (ROC) analysis. Variables significantly associated with DGF were identified by univariate logistic regression analysis. Multivariate analyses were performed using the logistic regression analysis posterior Wald test.
